# A Comparative Study on Rapid Wastewater Treatment Response to Refugee Crises

**DOI:** 10.1002/gch2.201800039

**Published:** 2018-10-23

**Authors:** Heta Kosonen, Amy Kim, Heidi Gough, Anna Mikola, Riku Vahala

**Affiliations:** ^1^ Department of Civil and Environmental Engineering University of Washington P.O. Box 352700 Seattle WA 98198‐2700 USA; ^2^ School of Environmental and Forest Sciences University of Washington P.O. Box 352100 Seattle WA 98198‐2700 USA; ^3^ Department of Built Environment Aalto University P.O. Box 15200 Aalto FI‐00076 Espoo Finland

**Keywords:** biological wastewater treatment, disaster response, project delivery, refugee crises, WWTS operation

## Abstract

Large‐scale population displacement can overwhelm wastewater treatment facilities and increase environmental pollution in the host communities. Academic research has discussed features that improve wastewater treatment systems' resiliency toward other types of disasters and rapidly changing operation conditions. Concepts that contribute to successful startup, refurbishment, and operation of biological treatment systems during refugee responses are yet to be identified. This study takes a novel approach to analyzing wastewater treatment system resiliency by presenting an input–mediator–output model analysis on advanced wastewater treatment delivery during refugee response in Jordan and Finland in 2015–2016. By comparing two distinctively different case studies, the research identifies principles that contribute to timely refugee response in advanced wastewater treatment systems on the dimensions of human resources, project environment, and wastewater treatment technology. These principles include 1) clear role division between agencies and stakeholders, 2) improving “human capacity” for rapid response decisions, 3) selecting a process that fits the regulative and operational environment, 4) enabling direct and fast information sharing, and 5) establishing fast‐track permitting processes for disaster conditions. Wastewater treatment system operators, regulative authorities, and aid organizations can use these findings to support rapid decision‐making in future disaster response situations.

## 1. Introduction

Wastewater management and treatment are critical during disaster response for ensuring the protection of human health and minimizing long‐term environmental consequences for the host community.^1^, ^2^ Empirical research and lessons learnt from prior disaster response events have resulted in guidelines that help responders with identifying the needed sanitation services,^3^ selecting the most suitable sanitation systems,^1^, ^4^, ^5^, ^6^ and organizing multi‐sectoral stakeholder activities.^7^, ^8^ However, these guidelines place little focus on the challenges faced during operational phases after the establishment of the sanitation systems. Especially, directions on emergency operations for modern wastewater treatment systems (WWTS) are missing.

In recent years, academic research has addressed disaster risk mitigation and preparedness of urban water systems through the concept of “resiliency.” A growing body of literature has started to define the features of resilient urban wastewater systems, i.e., systems that are able to minimize the magnitude and duration of disruptive events and adapt to changing conditions.^9^, ^10^, ^11^, ^12^ Studies to date have focused on the response to natural phenomena (e.g., extreme weather events leading to influent flow variation) and equipment failures (e.g., power outages, aging infrastructure, and mechanical issues).^11^, ^13^, ^14^ Political and secondary disasters, such as large‐scale population displacement due to natural disasters or political conflicts, have received less attention. Given the specific issues related to population displacement scenarios—such as the undefined length^15^ and the multi‐sectoral, highly political decision‐making environment^16^, ^17^—there is a need to identify features that increase WWTS resilience toward population‐displacement scenarios. Experiences from the aftermath of natural and humanitarian catastrophes, such as Hurricane Katrina and Syrian refugee crisis, have already demonstrated the potential for severe environmental impacts when displaced populations overwhelm the biological wastewater treatment facilities in host communities.^18^, ^19^

This study takes a novel approach to analyzing wastewater treatment system resiliency by presenting an input–mediator–output (IMO) model analysis on empirical data on advanced wastewater treatment delivery during refugee response in Jordan and Finland. IMO models are widely used in research investigating team decisions, processes, and productivity,^20^, ^21^ but have not been previously applied in the context of wastewater treatment. IMO model describes the wastewater treatment delivery process through “requirements of the environment” (inputs) that become “products for the environment” (outputs) through processes or different stages (mediators). By comparing two distinctively different case studies, i.e., applying a polar comparison method,^22^ this research takes initial steps in identifying a set of global stressors in WWTS operation during disaster response. Additionally, it proposes factors that contribute to a successful startup, refurbishment, and operation of biological treatment systems during disaster response. The findings of the study are applicable for improving resiliency and developing disaster response capacity in biological wastewater treatment system operation regarding human resources, project environment, and treatment technology. Wastewater treatment system operators, regulative authorities, and aid organizations can use the given recommendations to support rapid response decision‐making in future disaster response.

## 2. Background

The Syrian Conflict has led to the displacement of over 11 million people both internally and internationally.^23^ Ever since the beginning of the Syrian civil war in 2011, the Hashemite Kingdom of Jordan bordering Syria has been one of the countries hosting the largest number of Syrian refugees in relation to its national population.^24^ In 2015, the “Syrian refugee crisis” became a worldwide topic as the number of people seeking for asylum in the European Union exploded unexpectedly and sparked an international crisis as countries tried to cope with the flux of people and provide shelter and basic services for everyone.^17^ One of the countries receiving tens of thousands of migrants over a few months was Finland.^25^ The case study countries Finland and Jordan, the scale of their refugee response operations, as well as the technical specifications of the wastewater treatment systems examined in this study are introduced in **Table** **1**.

**Table 1:** Finland and Jordan in numbers regarding water, sanitation, and hygiene (WASH), and refugee response

Water supply and services		
	Jordan	Finland
Access to safe drinking water servicea)	93% of the population	97% of the population
Access to safe sanitation services^a^,b)	77% of the population	92% of the population
Renewable freshwater per capita (m^3^ per year)a)	77 m^3^	19 592 m^3^
Refugee response		
	Jordan	Finland
Refugee response	Long‐term (>5 years)	Short‐term (<0.5 years)
Number of registered asylum seekers^b^,c)	650 000	32 476
Refugee accommodation	Host community (84%) Refugee camps (16%)	Refugee centers (100%)

^a)^ World Bank (2018). “World Bank Open Data” https://data.worldbank.org/

^b)^ UNHCR, The UN Refugee Agency (2016). “UNHCR Syria Regional Refugee Response” https://data.unhcr.org/syrianrefugees/country/php?id = 107 (10/9, 2016)

^c)^ The Finnish Ministry of the Interior (2017). “Pakolainen pakenee vainoa kotimaassaan” http://intermin.fi/maahanmuutto/turvapaikanhakijat‐ja‐pakolaiset.

### 2.1. Case Study 1: Azraq Wastewater Treatment Plant

The Azraq refugee camp in Northern Jordan (established in 2014) is one of the five official settlements that have hosted Syrian refugees in Jordan since 2011. The Azraq camp is located in a remote area in the middle of Jordanian desert, where temperature changes are substantial and access is restricted. With the designed capacity to serve as a temporary home for up to 130 000 refugees, it is the second largest temporary housing settlement in the country. Azraq is often referred to as the “model refugee camp,” as its facilities were designed to overcome problems that Zaatari refugee camp and other refugee camps around the world have experienced.^26^

Among Azraq's improved facilities is its WWTS that is one of the first in the world to provide advanced treatment in a refugee camp setting. The wastewater treatment process is a moving bed biofilm reactor (MBBR) process with biological pretreatment and postchlorination. The initial design capacity of the treatment process was 400 m^3^ of wastewater per day, with an expected biological oxygen demand (BOD) load of 800 kg d^−1^. To comply with the Jordanian wastewater treatment regulations, the treated effluent from the system would have to have a BOD concentration of <60 mg L^−1^, a total suspended solids (TSS) concentration of <60 mg L^−1^, and nitrate (NO_3_‐N) concentration of <45 mg L^−1^.^27^

### 2.2. Case Study 2: Wastewater Treatment at Finnish Refugee Centers

The refugees and asylum seekers that arrived in Finland during the winter of 2015–2016 stayed in refugee centers that were established rapidly in existing, often underused facilities, such as camp centers and old school buildings that were easy to empty with a rapid schedule. Many of these buildings were located in remote areas and were for that reason not connected to centralized public utility services. Instead, the facilities treated their wastewater in small de‐centralized biological wastewater treatment plants that served the facilities and their immediate neighboring estates. This study focuses on three refugee centers in Southern Finland with wastewater treatment system design flows of 20, 30, and 58 m^3^ d^−1^. The expected BOD loads for the activated sludge systems with chemical pre‐precipitation were 9.1, 10, and 14 kg d^−1^, respectively. To comply with the Finnish wastewater treatment regulations, the treated effluent from the WWTS would have to have a BOD concentration of <15 mg L^−1^. Regulations for total phosphorus concentration vary between <0.7 and 1.0 mg L^−1^ depending on the WWTS.

## 3. Comparative Analysis Findings: Commonalities and Distinctions

This section discusses the commonalities and distinctions in wastewater treatment‐related decision processes in Finland and Jordan. The first subchapter describes the decision inputs, i.e., the state of the matters when wastewater treatment began. The second subchapter describes the processes and concepts that facilitated or hindered stakeholders in amending the situation in the beginning into functioning wastewater treatment systems, and the third subchapter summarizes the outcomes of the whole process regarding accomplishments and lessons learned.

**Figure 1:**
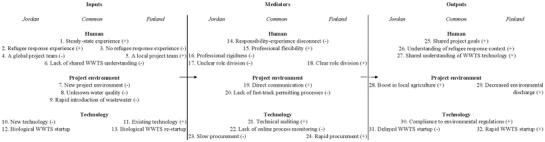
Comparative analysis of wastewater treatment response during refugee crisis in Jordan and Finland.

IMO and other forms of input‐process‐output models are typically illustrated as three‐column diagrams, where inputs are on the left side, mediators in the middle and the outputs on the right. **Figure** **1** represents the results of this analysis in the same format. Each input, mediator, and output of the decision process that, according to the interviewed stakeholders, demonstrated a positive impact on wastewater treatment delivery during refugee response is marked with a (+) sign. Respectively, those inputs, mediators, and outputs that hindered or delayed wastewater treatment delivery during refugee response are marked with a (‐) sign. Other features that were neither positive nor negative are not given any sign.

### 3.1. Decision Process Inputs

#### 3.1.1. Human

In both countries, stakeholders had steady‐state experience (Figure 1, #1) from WWTS management. In Jordan, where refugees had started to arrive already in 2011, some stakeholders had also experience with refugee response (Figure 1, #2). In Finland, all stakeholders were new to refugee response context (Figure 1, #3).

In the Azraq refugee camp, the global project team (Figure 1, #4) represented different nationalities and working cultures and had to thus adapt to each other's different norms and practices. In Finland, all stakeholders were local (Figure 1, #5), but the project team included stakeholders with no prior exposure to wastewater treatment‐related decision‐making and WWTS management. The lack of shared understanding of WWTS (Figure 1, #6) was also experienced in the Azraq camp, although all stakeholder groups consisted of individuals with prior exposure to wastewater treatment. This was mainly due to the modular MBBR technology not being previously used in Jordan, and the fact that other decision‐makers did not share a technical understanding of its specifications with the consultants.

#### 3.1.2. Project Environment

The project environment (Figure 1, #7) posed new challenges to the stakeholders that were involved in wastewater treatment‐related decision‐making in both countries. At the startup, stakeholders did not have adequate information on wastewater quantity and characteristics (Figure 1, #8). In Finland, the influent wastewater flow increased drastically overnight, but without a functioning flow meter, it was difficult to discern the amount of flow. Additionally, the stakeholders had limited information of the characteristics of the water, as water quality had only been sampled by quarterly grab samples and no real‐time online quality monitoring was available at the WWTS. In Azraq, the stakeholders had to deal with the fact that the wastewater treatment system was designed for different influent wastewater characteristics and flow patterns than was experienced during the treatment system startup. Before being hauled to the Azraq WWTS, the wastewater was stored in septic tanks for up to three weeks both during startup and regular operation. This was enough time for the wastewater to become anoxic. Moreover, the organic load of the influent wastewater was much higher than in the Zaatari refugee camp, which had been used as the base point for the process design.

The rapid introduction of wastewater (Figure 1, #9) was a common challenge in Finland and Jordan at the beginning of the refugee response. In Finland, the sudden change in influent flow to the WWTS was a direct result of the rushed timeline in establishing refugee centers. In Azraq WWTS, the rapid introduction of the raw wastewater was instead regulation driven: the consultants overseeing the startup process would have preferred first to fill the process with diluted wastewater and increase the concentrations gradually while the microbial communities in the aerated process compartments were building up. However, as this procedure was not compliant with the Jordanian wastewater treatment regulations, the whole treatment system was filled with untreated sewage with the intention to build microbial activity through sludge circulation.

#### 3.1.3. Technology

The modular MBBR units in Azraq WWTS had never been operated in similar conditions. The remote location, limited access to resources, and extreme temperatures put both the applied MBBR technology as well as the consulting and manufacturer company delivering the technology in a new project environment (Figure 1, #10). The treatment process was started up from the beginning with no seed sludge, which slowed down the activated sludge build‐up (Figure 1, #12). In Finland, the WWTS were already in operation at the beginning of the refugee response (Figure 1, #11). However, the combination of a rapid increase in influent hydraulic and organic load and malfunctioning aeration equipment had diminished the existing microbial communities that had been nitrifying in slow rates under the previously carbon‐limited conditions and the processes needed to be re‐started (Figure 1, #13).

### 3.2. Decision Mediators

#### 3.2.1. Human

In both Finland and Jordan, stakeholders involved in wastewater treatment‐related decision‐making experienced an internal conflict between their professional experience and responsibilities during the crisis (Figure 1, #14). In Finland, the real estate owners had to intervene with no prior experience with procurement decision during WWTS refurbishment. In Jordan, WWTS operators without the necessary MBBR technical familiarity had to advise on operational recommendations remotely. The adoption of new roles was facilitated by all stakeholders' professional flexibility (Figure 1, #15), i.e., willingness to accept tasks beyond their regular responsibilities and working extended hours to obtain new knowledge related to the startup of biological wastewater treatment systems. Stakeholders in both countries also perceived that the flexibility (Figure 1, #16), or the lack thereof, in regulation interpretation was beneficial for wastewater treatment delivery. Many saw this as the reason why the Finnish WWTS were reaching their permit requirements within a couple of months of the beginning of refugee response, and why Azraq WWTS was minimally achieving its effluent quality requirements with one‐third of its full capacity nine months after the first startup. Additionally, the unclear role division between Azraq WWTS project stakeholders contributed to the slow progress of the startup process (Figure 1, #17). In Finland, stakeholder roles were quickly defined and divided, which facilitated the rapid startup of the Finnish WWTS (Figure 1, #18).

#### 3.2.2. Project Environment

Frequent and direct communication facilitated decisions on biological wastewater treatment process operation in Jordan and Finland (Figure 1, #19). In Finland, “open lines of communication” were established by regulatory authority's initiative in the very beginning of the refugee response situation, but in the Azraq WWTS project, the establishment of direct and frequent communication took time. In addition to helping with clarifying stakeholder roles and responsibilities, direct and frequent communication was essential in resolving issues related to contractual and regulation obscurity in both countries that was caused by the need for rapid response to refugees' needs. In both countries, stakeholders mentioned the lack of fast‐track, disaster response suitable, permit processes as one of the mediating factors that challenged timely wastewater treatment delivery (Figure 1, #20).

#### 3.2.3. Technology

The successful startup of the biological treatment processes required systematic technical auditing of the process equipment in both countries (Figure 1, #21). As wastewater treatment plants in Finland were already in operation when the refugee response began, the auditing was conducted as one of the first activities. In Azraq, the treatment process equipment was brand new, so there was no need for assessing its condition at the beginning of the refugee response. Only when two startup attempts had already failed, stakeholders started to suspect the buildup of biological activity was repeatedly failing due to the poor condition of mechanical equipment. The extent of the refurbishment need came as a surprise both in Finland and Azraq, as the inadequately functioning, or completely nonexisting, monitoring equipment prevented stakeholders from understanding the “state of the process” through water quality characteristics (Figure 1, #22). The Finnish WWTS were refurbished quickly: first process parts were replaced less than two months after the refugee centers had started their operation. The rapid reconstruction was possible because the replaced process parts were standard wastewater treatment equipment, and the consultant leading the auditing and refurbishment knew local vendors and manufacturers and was able to finalize purchase decisions through an email or phone confirmation from the real estate owner (Figure 1, #24). In Azraq, the refurbishment was not as easy due to the remote location of the camp and the limited availability of the specific process parts needed for the MBBR process refurbishment. Also, the refurbishment process was delayed by the slow non‐governmental organization (NGO) procurement processes (Figure 1, #23) that, according to one of the project consultants, added an extra three months to any activity.

### 3.3. Decision Outputs

The biological wastewater treatment plants in the Azraq refugee camp and Finnish refugee centers were eventually operating according to the environmental regulations (Figure 1, #30). In Finland, this happened within a few months from the beginning of the refugee response (Figure 1, #32), while in Azraq, the WWTS was operational 12 months after the initially scheduled startup (Figure 1, #31). In both cases, the successful wastewater treatment plant operation required stakeholders sharing an understanding of the project goals (Figure 1, #25), the refugee response context (Figure 1, #26), and the limitations and capabilities of the applied treatment technology (Figure 1, #27). In Finland, the shared goals were established in the very beginning of the project, whereas in Azraq the stakeholders developed a shared understanding of the project goals after two startup attempts had already failed. Respectively, the WWTS in Finland were operating according to the regulations sooner than the WWTS in Azraq.

Contrary to the public reservations, the refugee response activities in Finland ended up decreasing, not increasing, wastewater‐related environmental pollution in the communities that were hosting refugee centers (Figure 1, #29). The treatment results in the small WWTS improved regarding effluent BOD, suspended solids, and nutrient concentrations, mainly as a result of treatment equipment refurbishment and optimized process operation and all studied systems adhered to the requirements of their environmental operation permits. In Azraq, the establishment of successful wastewater treatment operation on‐site enabled agricultural wastewater reuse creating potential new income opportunities for the camp residents and a boost in the local microeconomy (Figure 1, #28).

## 4. Implications

The comparative study on biological wastewater treatment startup in Finland and Jordan during refugee response revealed commonalities and distinctions between the stakeholder decision processes. Interestingly, while the refugee response context was very different in the two compared cases (Table 1), the starting points for wastewater‐related decision‐making shared many characteristics on human, project environment, and technology dimensions. In both countries, stakeholders had limited experience from disaster response and were operating in a new project environment with limited knowledge on wastewater quality and loading patterns. Biological process activity had to be built from the beginning, which in Finland meant re‐starting the processes and in Azraq, building up activity to the aerated MBBR tanks without seed sludge. In both situations, the process for enhancing microbial activity was limited by 1) lack of adequate data on water quality, 2) organic shock loading, and consequently 3) process operators' limited understanding of the biological treatment process growth kinetics under these types of extreme conditions. The decision mediators, such as clear role division in Finland and unclear role division in Jordan, were on the contrary distinctively different and led to different outcomes in wastewater treatment delivery. In Jordan, the operation of the activated sludge process was delayed by several months, and treatment requirements were only partially fulfilled by the end of the study period even when the hydraulic and organic loadings stayed lower than the design values. Comparably, the studied activated sludge processes in Finland were operating with improved treatment results within two months of the beginning of the refugee response, with influent hydraulic and organic loads that exceeded the design capacity. While the generalizability of the results of the polar case study comparison is limited, these emerging findings suggest that contextual inputs, such as the scale of refugee response, are not as crucial in determining the quality of wastewater treatment as the mediating processes and structures in decision‐making are. Based on these findings, we distinguished five principles that contribute to timely refugee response in advanced wastewater treatment systems on the dimensions of human agency, project environment, and wastewater treatment technology. These principles are 1) clear role division between agencies and stakeholders, 2) improving “human capacity” for rapid response decisions, 3) selecting a process that fits the regulative and operational environment, 4) enabling direct and fast information sharing, and 5) establishing fast‐track permitting processes for disaster conditions. The findings of this study can be applied to improve the resiliency of wastewater treatment in the face of disaster and emergencies, such as the studied case of global refugee crisis resulting from the Syrian civil war.

### 4.1. Clear Role Division between Agencies and Stakeholders

Empirical evidence from Azraq and Finland suggests that clear role division facilitated interdisciplinary teams' response to the refugee crisis and helped them in continuing wastewater treatment without interruptions. In Finland, where roles were well defined from the beginning and stakeholders quickly developed mutual goals, wastewater treatment plants were operating according to the environmental permit requirements within two to three months from the beginning of the refugee response. In Azraq, where stakeholders' mutual understanding of roles and goals took more time to develop, wastewater treatment process startup was delayed by several months. The findings align with prior results from construction management research that have time and again defined the clear definition of responsibilities and roles as one of the critical success factors for project delivery in various contexts.^28^, ^29^, ^30^, ^31^ However, there are limited recommendations on how to achieve this in practice. “Selection of team leader” and “clear division of roles and responsibilities among team members” are also mentioned as two main considerations in the WHO guideline for sanitation safety planning (SSP),^32^ but none of the interviewed stakeholders mentioned these plans as a resource that was used in team organization. A possible reason for the lack of SSP or guideline usage during crisis response is that these plans, too, are too general to guide decision‐making in an advanced wastewater treatment process startup. Consequently, we propose that specific guidelines for communication and decision procedures during the crisis operation and startup of advanced wastewater treatment systems would be added to the country‐specific SSPs. These guidelines could include detailed examples and suggestions including, but not be limited to, 1) the party that initiates the assessment on wastewater treatment systems' capacity to respond to changed conditions, 2) who needs to know what (e.g., do the regulatory agency or NGO employees need to understand the technology, and to what extent do operators need to understand biological wastewater treatment or project goals), and lastly 3) who is an “active decision‐maker,”, i.e., people that are making decisions in the field, and who is in an advisory role.

### 4.2. Improving “Human Capacity” for Rapid Response Organization and Decision‐Making

In addition to research on “human agency” in emergency response wastewater treatment in team level, our preliminary findings suggest that more research is needed to understand better how wastewater treatment experts' personal, individual capacities could be improved to better align with the type of actions that are needed during crisis response. Both in Finland and in Azraq, decision‐making was facilitated and accelerated by individuals who were strongly driven by their motivation to protect the environment and helping the refugees. In Finland, the governmental authority took a leading role in the very beginning of the refugee response and sought creative solutions to technical problems in collaboration with other stakeholders. In Azraq, stakeholders started to reach a mutual understanding of the project goals after two startup failures, when aid organization employees and technical consultants tightened their collaboration and took strong initiative on solving communication issues with the governmental authorities. Overall, wastewater treatment delivery was facilitated by professional flexibility and hindered by professional rigidness. These findings are supported by prior research on disaster response teams that has discovered that procedural and structural innovation and ability to take on different roles is necessary for successful crisis operations.^33^, ^34^ They also align with Belbin's team role theory^35^ and related research on team development in various work environments. This research suggests that teams that are under continuous change are best supported and led by individuals that display the innovative characteristics of “resource investigators,” “plants,” and “shapers.”^36^ In the studied emergency situations, teams were emergent, as they were formed out of necessity, their composition was new, and they were dealing with new nonregular tasks.^37^ Still, in both cases, the leaders with “shaper” and “plant” capacities emerged and naturally claimed their role. Furthermore, the presence of a “specialist,” another one of the Belbin team roles, was essential for successful plant operation both in Finland and Azraq. According to Belbin,^35^ the team roles, except for the role of a “specialist” which develops through experience and knowledge, are strongly defined by individual characteristics. Thus, to facilitate future disaster response, we suggest that wastewater treatment professionals should be trained to understand and identify their characteristics, how they operate in team environments, and how they make rapid decisions in disaster response conditions. Additionally, it would be beneficial for each community, country, or emergency response unit to develop a roster of trusted “specialists” with extensive wastewater treatment expertise, who would guide stakeholder decision‐making in wastewater treatment‐related issues in future emergency situations. Capacity building for more effective response activities could be done through team role improvisation trainings that help stakeholders prepare for selecting alternative courses of action and taking on new roles,^38^, ^39^ or through Belbin team role training or similar systems that allow individuals to define their strenghts and learn to communicate and coordinate with other team members. For decision‐making under high stress, wastewater treatment professionals could be prepared by enhancing their understanding of judgments under stress.^40^, ^41^

### 4.3. Selecting a Process that Fits the Regulative and Operation Environment

Recent research on advanced wastewater treatment systems' capacity to endure dynamic organic loads has shown that MBBRs and other attached growth systems are an ideal choice for conditions, where the influent organic load can change rapidly.^42^, ^43^, ^44^ However, at the Azraq refugee camp, the MBBR system startup failed, not due to system capacity related issues, but due to a combination of factors related to stakeholder communication, obscurity in regulations, lack of process monitoring, and slow procurement and refurbishment processes. Academic literature on wastewater treatment in extreme loading conditions has so far provided very limited insights on how operable advanced treatment systems, such as MBBRs and other attached growth systems, in fact, are in dynamic organic load conditions. To our knowledge, there are few studies that have investigated the adaptation of these technologies in field conditions, as most studies have focused on testing extreme conditions in laboratory scale.^42^, ^43^ Our findings provide preliminary evidence that in emergency conditions, the most optimal choices for wastewater treatment system are those that fit the regulative and operation environment. The biological performance under dynamic loading conditions is subsidiary, if treatment system startup is primarily limited by discharge regulations, slowness in procurement, or stakeholders' inability to react promptly to changes in the operation environment. In Finland, stakeholder response was quick, as everyone making technical decisions was familiar with activated sludge technology, regulations for treated effluent were already set in place, and system refurbishment could be done with standard equipment that was easy to procure. In Jordan, the MBBR system was new to operators and regulatory authorities, which complicated both on‐site decision‐making and regulatory decision‐making, and ultimately delayed process startup. For instance, the Jordanian wastewater treatment regulations prohibited process startup with seed sludge and as a result, the project team had to rely on the slower option of building up biological activity through recycling. Furthermore, procurement was difficult in Jordan, as the needed special process parts were not readily available within the country. We propose that assessments on wastewater treatment resiliency not only address treatment process' ability to maintain sufficient levels of BOD and nutrient removal in dynamic conditions (e.g., based on prior research or operation data), but also include equal consideration of system vulnerability in terms of restart and repair capacity (availability of seed sludge, process parts, and equipment with short notice) and expertise availability (general conspicuousness of the treatment system in the country, how likely are stakeholders to understand this system, and how easy is it to get expert consultancy with short notice) in the operation context. These suggestions complement the wastewater treatment system selection criteria that have been previously presented for refugee settlements (1) and more generally for flexible water and wastewater designs. They also provide empirical evidence on the previously identified necessity of modularity, redundancy for resilient wastewater treatment provision in disaster conditions,^14^, ^45^, ^46^ and complement the previously identified set of selection criteria by introducing “expertise availability” as a key criterion for resilient wastewater treatment systems for refugee response.

### 4.4. Enabling Direct and Fast Information Sharing

The polar comparison displayed the importance of face‐to‐face meetings and direct communication in successful stakeholder coordination and wastewater treatment delivery during refugee response. The findings are aligned with prior research that has also recognized the need for “hi‐touch” communication and direct and information interaction in dynamic project environments where conditions and team composition are changing.^47^, ^48^ Consequently, we recommend that the establishment of “open lines of communication” between all stakeholders is prioritized whenever wastewater treatment systems are brought online in similar conditions. Depending on the regulative and operation environment, the transparent and inclusive communication practices should include a set of different activities ranging from face‐to‐face meetings to online communication and real‐time information sharing systems. In Finland, the physical proximity of all stakeholders facilitated information sharing as communication was possible through phone conversations and all stakeholders were able to conduct site visits to the WWTS whenever it was needed. For the international team of Azraq WWTS stakeholders, the opportunities for real‐time information sharing were more limited: the consultants were supposed to provide operational support remotely, but as the remote process monitoring and operation technology were never functional due to the lack of necessary information and communications technology (ICT) infrastructure, this was not possible. While “flatness” of communication has been previously identified as a feature that enhances wastewater treatment systems recovery speed after sudden perturbations,^49^ academic research has not discussed the role of monitoring technology and data sharing systems in increasing the resiliency of wastewater treatment systems.^50^ Additionally, while tools have been developed for information sharing during disaster response^51^, ^52^ and for WWTS operation during complex scenarios,^6^, ^53^ there is little empirical evidence of their applicability to disaster response. With our preliminary findings, we call for more case studies to better understand the opportunities and limitations of remote monitoring and online data management platform use in wastewater treatment plant startup and operation during disaster response. The evidence from Finland and Jordan suggests that openly accessible process monitoring data and frequent all‐stakeholder discussions about their implications are the key to successful and timely disaster response in wastewater treatment plants.

### 4.5. Fast‐Track Permitting Processes for Disaster Conditions

Both in Finland and in Azraq, inflexible permitting processes were hindering timely response to the refugee crisis in wastewater treatment plants. In Azraq, the MBBR startup plan was completely prevented by strict regulations that did not allow discharge from wastewater treatment plants until the required nitrification and BOD removal rates had been achieved in full scale. In Finland, slow environmental permitting process prevented quick reactions to increased influent organic load as any change in existing WWTS configuration would have required environmental permit renewal. Capacity increase was made possible only by regulative authority's deep understanding of the Finnish environmental permits and wastewater treatment, and consequently its ability to come up with creative regulative solutions. To ensure uninterrupted wastewater treatment in the future, global guidelines for permitting and controlling “emergency operation” in municipal wastewater treatment plants would be needed. Without these, the resiliency of the wastewater treatment system itself becomes irrelevant as was seen in Azraq WWTS: MBBR treatment process was a well‐justified choice for a dynamic influent loading pattern, but since regulation restrictions prevented the system from functioning in the first place, response to dynamic wastewater load conditions was not possible for the first six months of the WWTS operation. Prior literature on wastewater treatment resiliency has only introduced a limited discussion on the role of legislation or permitting in preventing or enabling the resiliency of the wastewater system.^10^, ^50^ In their review of the “state of the art” in wastewater treatment resiliency research, Juan‐García et al.^50^ listed all interventions that scholars had suggested for improving resiliency of wastewater systems. None of these were related to the permitting processes or environmental legislation. In another study evaluating the state of wastewater treatment resiliency, Johannessen and Wamsler^10^ identified enabling and disabling factors that influence the different resilience levels. While environmental permitting issues were not directly addressed, “power games and political self‐interest” and “lack of financial and other resources to handle beyond normal” were identified as factors disabling resiliency in wastewater treatment and “interinstitutional coordination” and “microgovernance arrangements” as factors enabling resiliency.

Another context where the need for expedited environmental permitting processes could have, but so far has not, naturally emerged is the scholarly discussion on European refugee crisis and its policy implications. While active and extensive, this conversation has for now focused on international regulation of the movement of people and sharing of financial responsibilities,^54^ not on direct implications of the rapid population increases in environmental policies at the host community level. The empirical evidence presented in this study suggests that flexible regulation is a requirement for resilient wastewater treatment during disasters. Future efforts in both academia and practice should aim to find ways to adjust wastewater‐related policies and permits, and their development to better fit situations of rapid response on a regional and local level without increasing the burden of disaster to the environment. References for this work could be found from numerous case studies around the world, where cities and communities have adapted expedited emergency permit processes to arrange emergency housing^55^ or to avoid environmental catastrophes, such as oil spills.^56^

## 5. Experimental Section

*Data Collection*: Data were collected through interviews with 21 individuals. Altogether 24 interviews were recorded as some individuals were interviewed more than once. All interviewees were involved with wastewater treatment delivery at the Azraq refugee camp in Jordan or at the three refugee centers in Southern Finland. Interviews were conducted face‐to‐face or via telephone between January and September in 2016. In Jordan, two of the interviews were conducted in Arabic, two interviews in English with Arabic assistance, and the remaining eight interviews in English. In Finland, all eight interviews were conducted in Finnish. The translators, all native Arabic or Finnish speakers, were part of the research group and had a strong technical background in water and environmental technology. **Table** **2** summarizes the details about interviewees.

**Table 2:** Interview details

Azraq refugee camp – Jordan							
	Interviewee job title	Role in WWTS project	Work location	Related work experience	Education	Experience with emergency response	Language
Technical consultants	Technical sales	Involved in early design and installation, providing operators and project owners with consultancy on process related and other technical issues, head of wastewater treatment process startup procedures	Mainly off‐site and abroad, occasional visits to the WWTS	6 years	Master in Science (MS)	No	English
	Project manager	Head of wastewater treatment process design, involved in design and operation throughout the project, coordinating technical and operational changes with operators and project owners, consulting operators and project owners	Mainly off‐site and abroad, occasional visits to the WWTS	15 years	Bachelor in Science (BS)	No	English
Contractors	WWTS operator 1	Responsible for wastewater treatment process operation on‐site, monitoring process performance, testing water quality and implementing operational changes when needed	On‐site daily	>20 years	Undergoing BS	No	English and Arabic
	WWTS operator 2		On‐site daily	>20 years	Vocational training	No	Arabic
	WWTS operator 3		On‐site daily	5 years	No training/education	No	Arabic
	Construction manager	Project manager overseeing the construction of the wastewater treatment plant, representative of the contractor working for the consultant, communicating project delivery related issues with consultants and aid organization	Mainly off‐site but within the country, visits to WWTS when needed	16 years	BS	No	English
NGO employees	WASH officer 1	Coordinating WASH initiatives in refugee camps and host communities. Liaison between all project stakeholders and representative of the project owner	Working in several locations, visiting WWTS several times a month	12 years	MS	Y	English
	WASH officer 2		Mainly located in the NGO headquarters	19 years	BS	Y	English
	WASH officer 3		Working in several locations, visiting WWTS several times a month	Yes	BS	Y	English
	WASH consultant	Outside consultant brought into the project to facilitate process re‐configuration and technical decision‐making related to the biological treatment process	Working in the Azraq refugee camp	>20 years	BS	Y	English
Finnish refugee centers – Finland							
	Interviewee job title	Role in WWTS project	Work location	Related work experience	Education	Experience with emergency response	Language
Consultants	Consultant 1	Head of wastewater treatment process design, involved in design and operation throughout the project, coordinating technical and operational changes with operators and project owners, consulting operators and project owners	Working in several locations, visiting WWTS several times a month	5 years	MS	No	Finnish
	Consultant 2	Responsible for regulatory wastewater sampling procedure planning and analyses	Mainly off‐site, monthly visits to WWTS	>20 years	MS	No	Finnish
Contractors	WWTS operator/consultant	Responsible for wastewater treatment process operation on‐site, monitoring process performance, testing water quality, and implementing operational changes when needed	Working in several locations, visiting WWTS several times a week	>20 years	Vocational training	No	Finnish
	WWTS engineer		Working in several locations, visiting WWTS several times a week	4 years	MS	No	Finnish
NGO employees	Head of refugee center	Responsible for running the daily operations of the refugee center	Working at the refugee center	1 year	MS	No	Finnish
	Refugee center manager		Working at the refugee center	1 year	–	No	Finnish
Real estate owners	Real estate owner 1	Selecting contractors, responsible for making final decisions on procurement	Working in several locations, visiting WWTS when needed	1 year	–	No	Finnish
	Real estate owner 2		Mainly off‐site, no regular site visits to WWTS	1 year	–	No	Finnish
Government employees	Regulatory authority	Overseeing the compliance of WWTS environmental permits	Working in several locations, visiting WWTS when needed	>20 years	MS	No	Finnish

The topics that were covered included technical understanding, resources used for decision support, procedures in communication and decision‐making, and demographics. All questions were open‐ended and undirected. Before interviews, the questions were pilot tested with an expert in conducting mental model interviews. **Table** **3** summarizes the interview topics.

**Table 3:** Interview topics

1. Background	
Demographics	
Experience with wastewater treatment and emergency response	
2. The biological wastewater treatment system	
Explanation of the current system configuration	
3. Events	
Predelivery events	
	Stakeholder communication prior to construction
	WWTS delivery to the site
	Events during construction and assembly
	Events during initial process startup
Recent events	
	Events related to WWTS operation and construction
	Stakeholder communication during these events
	Decision processes and stakeholder roles
4. Resources	
List of resources, including personnel and technology, used during the decision‐making of the process	
5. Wastewater treatment system performance	
Issues with foaming, bulking, or other problems considered system upsets	
Impacts of process upsets	
6. Operation, maintenance, and decision‐making	
Procedures used when making operational changes	
Documentation of operational/assembly changes	
	The content of documents
	The use of documentation and data in decision‐making
Lessons learnt, expertise gained	

Face‐to‐face interviews were conducted at the interviewees' place of work (e.g., construction trailer office). Interviewers toured the wastewater treatment plants with the interviewees before or after the interviews. Observational information gained during conversations during tours, during site visits, and during interviews was documented in notebooks during post‐tour reflections. Observational and reflective data were used as secondary data to complement the primary interview data.

Interviewees were given an option to have the interviews recorded. All but three of the interviews were audio‐recorded and transcribed to text in the language they were conducted in. Hand‐written notes were used to document the three interviews that were not recorded.

**Figure 2:**
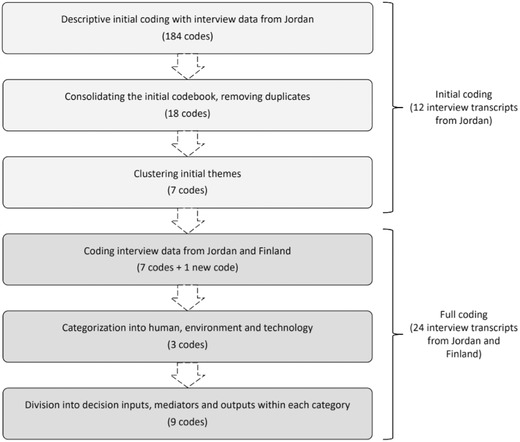
The qualitative coding process.

*Data Analysis*: The written narratives of the interview data were analyzed in two phases by using the Atlas.ti qualitative data analysis software as the analysis platform. **Figure** **2** summarizes the coding and data analysis process. In the first phase, researchers used conventional content analysis^57^ to identify factors that impacted stakeholders' mental models, i.e., thought process constructs, during wastewater treatment delivery at the Azraq camp. This analysis was based on 12 interviews that were conducted in the Azraq wastewater treatment plant in January–May 2016.^58^ The qualitative coding process revealed seven emerging themes, e.g., groups of concepts, which influenced stakeholder decision‐making processes during wastewater treatment plant construction and startup operation. The themes were further divided into contextual (physical location, resources, risk, and uncertainty) and internal (team dynamics, communication, personal characteristics, and experience and knowledge) based on whether or not they were dependent on individual stakeholders' or stakeholder groups' input.^57^

In the second phase of the data analysis, the internal and contextual concepts from the first phase were used as a “codebook” for analyzing the full data set of this study. Since all identified internal concepts were related to stakeholders' personal or group resources, the group was renamed as “human resources.” The contextual concept group was in turn re‐named as “project environment” as all concepts were related to the environment where stakeholders made decisions about wastewater treatment. Also, a new theme “process technology” was used for all excerpts where stakeholders described wastewater treatment technology and process performance. The coded excerpts were further classified into “decision process inputs,” “decision process mediators,” and “decision process outcomes” based on their role in the operational decision‐making process. For example, during the initial startup “new process technology” was classified as one of the decision process inputs and “the lack of inclusive communication” as one of the decision process mediators that led to the decision process outcome of “incomplete chemical oxygen demand (COD) and nitrogen removal.”

*Input–Mediator–Output Model*: Input–output models and their different variations, such as IMO model, have been widely used in research investigating team decisions, processes and productivity.^20^, ^21^ IMO models describe processes as “requirements of the environment” (inputs) that become “products for the environment” (outputs) through processes or different stages (mediators).^59^ In this study, the inputs of the IMO model were defined as the human resources, and project environment and treatment system features that existed at the beginning of the wastewater treatment delivery. Following the examples and definitions from prior studies,^59^ mediators were defined as the processes and structures through which stakeholders acted during wastewater treatment delivery. The outcomes of the IMO model describe the “state of the matters” at the end of the process, i.e., the wastewater treatment system performance and lessons learnt from the refugee response process.

*Polar Types Comparison*: This study used a polar type comparison method to identify commonalities and distinctions in the wastewater treatment and management decision‐making during refugee response in Jordan and Finland. The polar comparison is a standard method for case study research that targets new phenomena that have not been previously studied at large.^22^, ^60^ The idea was to create a baseline by examining contradicting attributes between two distinctively different, polar cases.^60^ The results of the comparison could then be used for defining general commonalities across a number of case studies; the logic is that if the polar examples share similarities, these similarities are expected to be shared with other cases that lie between the polar extremes.

Due to their distinctive differences presented in Table 1, Finland and Jordan are a polar comparison for wastewater treatment delivery practices during refugee response. As all interviewees were directly involved in operation and reconstruction of biological treatment systems during the global refugee crisis in 2015–2016, the study also represents cases with “high experience level of the phenomena,” which are considered the most appropriate for polar comparison by case study theory.^22^, ^60^

## Conflict of Interest

The authors declare no conflict of interest.
